# Stringent Response and AggR-Dependent Virulence Regulation in the Enteroaggregative *Escherichia coli* Strain 042

**DOI:** 10.3389/fmicb.2018.00717

**Published:** 2018-04-10

**Authors:** Mário Hüttener, Alejandro Prieto, Joan Espelt, Manuel Bernabeu, Antonio Juárez

**Affiliations:** ^1^Department of Genetics, Microbiology and Statistics, Faculty of Biology, University of Barcelona, Barcelona, Spain; ^2^Institute for Bioengineering of Catalonia, The Barcelona Institute of Science and Technology, Barcelona, Spain

**Keywords:** (p)ppGpp, AggR, EAEC, biofilm, AAF/II

## Abstract

Virulence expression in the enteroaggregative *Escherichia coli* strain 042 requires the transcriptional activator AggR. We show in this report that, as reported for other virulence factors, the nucleotide second messenger (p)ppGpp is needed for a high expression level of AggR. As expected from these findings, expression of AggR-activated genes such as the AafA pilin subunit is downregulated in the absence of (p)ppGpp. Considering the fact that biofilm formation in strain 042 requires the AafA protein, biofilm development in strain 042 is impaired in derivatives that lack either the AggR protein, the virulence plasmid that encodes AggR (pAA2) or the ability to synthesize (p)ppGpp. These results show a direct correlation between (p)ppGpp, expression of AggR and biofilm development in strain 042.

## Introduction

Enteroaggregative *Escherichia coli* (EAEC) strains are diarrheal pathogens (Kaper et al., 2004). The EAEC strains can be distinguished from enteropathogenic *E. coli* (EPEC) because of their different patterns of adherence to HEp-2 cells. Whereas EPEC display a “microcolony” pattern of adherence, EAEC display a characteristic aggregative or “stacked-brick” pattern (Nataro et al., 1987). EAEC adherence to intestinal cells is mediated by fimbrial adhesins, designated aggregative adherence fimbriae (AAFs).

Epidemiological studies have shown that EAEC strains are genetically heterogeneous. Hence, a large number of virulence factors have been identified in EAEC clinical isolates (Okeke et al., 2010). The strain O104:H4 is an example of EAEC genetic heterogeneity. It caused a few years ago in Germany a large outbreak of bloody diarrhea (Frank et al., 2011). Isolates from the O104:H4 outbreak harbor a plasmid (pAA) that encodes, among other virulence factors, the fimbriae that mediate the EAEC type of adherence (Bielaszewska et al., 2011). Nevertheless, unlike typical EAEC strains, strain O104:H4 contains a prophage encoding the Shiga toxin (Mayer et al., 2012), which is a well-characterized virulence determinant usually expressed by a different *E. coli* pathotype, enterohemorrhagic *E. coli* (Nataro and Kaper, 1998). The prototypical strain for the study of EAEC virulence factors and pathogenicity is strain 042, which caused diarrhea in a volunteer trial (Nataro et al., 1995). Its genome sequence is available (Chaudhuri et al., 2010) and the virulence factors are characterized. Strain 042 harbors the IncFIC virulence plasmid pAA2 (Nataro et al., 1985; Chaudhuri et al., 2010), which codes, among other genes, for the fimbrial adhesion determinants (the AAF/II variant of AAF), for the virulence master regulator AggR and for other virulence determinants (Nataro et al., 1994; Czeczulin et al., 1997; Chaudhuri et al., 2010; Morin et al., 2010). The AggR protein belongs to the family of the AraC-like transcriptional activators and is encoded by many EAEC strains. AggR activates it is own expression (Morin et al., 2010) and also the expression of several EAEC virulence factors encoded in the pAA2 plasmid (Morin et al., 2013), including the surface protein dispersin Aap (Sheikh et al., 2002) and the genes responsible for the synthesis of the AAF/II fimbriae (Elias et al., 1999). AggR also regulates the expression of some chromosomally encoded virulence determinants, such as a type VI secretion system identified in strain 042 (Morin et al., 2013). Details of *aggR* regulation are available. Regulation occurs both at the transcriptional and post-transcriptional levels. *aggR* transcription is repressed by the nucleoid associated protein H-NS (Morin et al., 2010). The FIS protein is also required for *aggR* expression (Sheikh et al., 2001). Post-transcriptional repression of AggR expression by the Aar protein (an AraC-member of negative regulators) has been recently described (Santiago et al., 2014).

The nucleotide second messenger (p)ppGpp drives the stringent response in bacteria (as reviewed in Dalebroux et al., 2010). This alarmone also plays relevant roles in bacterial persistence and virulence (Dalebroux et al., 2010; Maisonneuve et al., 2013). Classical examples of (p)ppGpp modulating bacterial virulence include, among others, adherence of enterohemorrhagic and uropathogenic *E. coli* strains (Aberg et al., 2006, 2008, 2009; Nakanishi et al., 2006) or invasion in *S. enterica* serovar Typhimurium (Pizarro-Cerdá and Tedin, 2004; Song et al., 2004). In this work we present new data regarding AggR regulation. We show that (p)ppGpp is required for proper expression of this transcriptional activator. According to this observation, the expression of AggR-activated virulence determinants and the formation of biofilm are significantly impaired in a (p)ppGpp^0^ mutant derivative of strain 042.

## Materials and Methods

### Bacterial Strains, Plasmids, and Culture Media

All bacterial strains and plasmids used in this work are listed in **Table 1**. Cultures were routinely grown in Luria Broth (LB) medium (10 g NaCl, 10 g tryptone, and 5 g yeast extract per liter) with vigorous shaking at 200 rpm (Innova 3100, New Brunswick Scientific). Antibiotics were used at the following concentrations: kanamycin (Km) (50 μg ml^-1^), chloramphenicol (Cm) (25 μg ml^-1^), carbenicillin (Cb) (100 μg ml^-1^).

**Table 1:** Bacterial strains and plasmids used in this study.

Bacterial strains	Description	Source or reference
042	*E. coli* EAEC, Cm^r^ Sm^r^ Tc^r^	Prof. I. Henderson
042LC	042 Δ*lacZ*Δ*cat*	This work
042LCaggRlacZ	042LC *aggR::lacZ*	This work
042LCaggRlacZrelA	042LC *aggR::lacZ*Δ*relA*	This work
042LCaggRlacZrelAspoT	042LC *aggR::lacZ*Δ*relA* Δ*spoT*	This work
042aggR	042 *aggR*::km	This work
042relAspoT	042 Δ*relA* Δ*spoT*	This work
042AggRFlag	042 AggR-Flag	This work
042AafAFlag	042 AafA-Flag	This work
042AggRFlagrelA	042 AggR-Flag Δ*relA*	This work
042AafAFlagrelA	042 AafA-Flag Δ*relA*	This work
042AggRFlagrelAspoT	042 AggR-Flag Δ*relA* Δ*spoT*	This work
042AafAFlagrelAspoT	042 AafA-Flag Δ*relA* Δ*spoT*	This work
042pAA^-^	042 Cured from pAA2 plasmid	This work
DH5α	*E. coli, fhuA2 lac(del)U169 phoA glnV44 Φ80′ lacZ(del)M15 gyrA96 recA1 relA1 endA1 thi-1 hsdR17*	Taylor et al., 1993

**Plasmids**	**Description**	**Source or reference**

pBAD18	rep_pMB1_ p_araBAD_ Cb^r^	Guzman et al., 1995
pBAD-AggR	pBAD18 + *aggR* from EAEC 042	This work
pBR322	ori_p_MB1, Tc^r^, Ap^r^	Bolivar et al., 1977
pBR322-SpoT	pBR322 + *spoT* from EAEC 042	This work
pKD3	oriRγ, Cm^r^, Ap^r^	Datsenko and Wanner, 2000
pSUB11	FLAG- and Km^r^-coding template vector	Uzzau et al., 2001
pKD4	oriRγ, Km^r^, Ap^r^	Datsenko and Wanner, 2000
pKD46	oriR101, repA101 (ts), AraBp-gam-bet-exo	Datsenko and Wanner, 2000
pCP20	λcI857 (ts), ts-rep (FLP ts)	Cherepanov and Wackernagel, 1995


To construct plasmids pBAD-AggR and pBR322-SpoT, the *aggR* and *spoT* genes from strain *E. coli* 042 were amplified using oligonucleotides AggREcoRI18.5-AggRXbaI18.3 and spoT042pbr322ECORIfw5-spoT042pbr322BAMHIrev3 (see **Supplementary Table S1**, for the sequence) together with Phusion Hot Start II High-Fidelity DNA Polymerase (Thermo Scientific) following the manufacturer’s recommendations. *aggR* and *spoT* amplification with the above referred oligonucleotides generates EcoRI/XbaI and EcoRI/BamHI sites flanking the *aggR* and *spoT* genes, respectively. The corresponding EcoRI/XbaI and EcoRI/BamHI fragments were cloned into the vector pBAD18 and pBR322 digested with the same enzymes. The resulting plasmid was termed pBAD-AggR and pBR322-SpoT.

### Genetic Manipulations

All enzymes used to perform standard molecular and genetic procedures were used according to the manufacturer’s recommendations. To introduce plasmids in *E. coli*, bacterial cells were grown until an OD_600__nm_ of 0.6. Cells were then washed several times with 10% glycerol, and the respective plasmids were electroporated by using an *Eppendorf* gene pulser (Electroporator 2510).

The WT 042 strain is Cm resistant (Cm^r^) and encodes the *lacZ* gene. To construct an *aggR::lacZ* transcriptional fusion in this strain we needed first to knock out the *lacZ* gene. Taking into account that Km resistance (Km^r^) is conferred by the genetic approach used to generate the *lacZ* transcriptional fusion, we needed a second marker for selection of additional mutations. Hence, we decided to inactivate the *cat* gene as well. Upon obtaining the *lacZ cat* derivative of strain 042 (termed 042LC) we used it to generate a transcriptional *lacZ* fusion on the *aggR* gene, generating strain 042LCaggRLacZ (*aggR::lacZ*).

Since inactivation of *relA* and *spoT* genes in strain 042 is a previous step to evaluate the role of (p)ppGpp on expression of the *aggR* gene, we knocked out both genes in strain 042 and in different mutant derivatives. To that end, we fist knocked out the *relA* gene in strains WT 042, 042LCaggRLacZ, 042AggRFlag, and 042AafAFlag. First, a Cm^r^ derivative was obtained by the λ Red recombinant method in the *relA* locus and then, we took advantage of FLP recombinase encoded by pCP20 plasmid to eliminate the Cm^r^ cassette, generating the corresponding Δ*relA* isogenic mutants. Thereafter, the *spoT* gene was deleted from the Δ*relA* derivatives of the different strains by the λ Red recombinant method inserting a Cm^r^ cassette in the *spoT* locus. Again, pCP20 plasmid was used to eliminate the Cm^r^ determinant, generating the Δ*relA*Δ*spoT* mutant derivative in the strains; WT 042 (042relAspoT), 042LCaggRLacZ (042LCaggRlacZrelAspoT), 042AggRFlag (042AggRFlagrelAspoT), and 042AafAFlag (042AafAFlagrelAspoT), respectively.

To obtain the above referred mutant derivatives lacking *lacZ, cat*, *aggR*, *relA*, and *spoT* alleles in the EAEC strain 042, the λ Red recombinant method described by Datsenko and Wanner (2000) was used. Briefly, the Km^r^ cassette of plasmid pKD4 was amplified using oligonucleotides LacZ042P1/LacZ042P2, Cat042P1/Cat042P2, and AggR042P1/AggR042P2 for *lacZ*, *cat*, and *aggR* deletions, respectively (see **Supplementary Table S1**, for the corresponding sequences). For mutation in the alleles *relA* and *spoT*, the Cm^r^ cassette from plasmid pKD3 was amplified using respectively oligonucleotides RelA042P1/RelA042P2 and SpoT042P1/SpoT042P2 (see **Supplementary Table S1**, for the corresponding sequences). DNA templates were treated with DpnI (Thermo Scientific) following manufacturer recommendations and then, purified and electroporated to the competent cells. Mutants were selected on LB plates containing the appropriate selection marker (Km or Cm) and the successful deletion of the corresponding gene was confirmed by PCR using the primers KT or Cat-C1 (Km^r^ and Cm^r^, respectively) in combination with specific primers located in the remaining gene sequence in the bacterial chromosome (see **Supplementary Table S1**, for the corresponding sequences).

When necessary, the antibiotic resistance cassette was eliminated by transforming the mutant strain with plasmid pCP20 and subsequent incubation at 42°C for two or more passages as reported (Datsenko and Wanner, 2000). The pCP20 plasmid encodes the Flp recombinase that catalyzes the recombination between the FRT sites flanking the antibiotic resistance cassettes (Cherepanov and Wackernagel, 1995). The FRT-generated site in the gene *aggR* was used to integrate plasmid pKG136 (Ellermeier et al., 2002), thereby generating the transcriptional *aggR*::*lacZY* fusion.

Insertions of FLAG sequences to the *aggR* and *aafA* genes were obtained by a modification of the λ Red recombinant method, as described by Uzzau et al. (2001). The antibiotic-resistance determinant of plasmid pSUB11 was amplified using oligonucleotides AggR3xP1/AggR3xP2 and AafA3xP1/AafA3xP2 for the *aggR* and *aafA* genes, respectively (see **Supplementary Table S1**, for the corresponding sequences). Mutants were selected on LB plates containing Km, and successful FLAG insertion was confirmed by PCR using the oligonucleotides KT (Km^r^) in combination with specific oligonucleotides located in the remaining gene sequence nearby (see **Supplementary Table S1**, 3xP1UP/3xP2DOWN series oligonucleotides). The chromosomal fusions AggR-Flag and AafA-Flag were constructed in the parental strain *E. coli* 042. The Δ*relA* and Δ*relA*Δ*spoT* mutations were introduced in the strains encoding Flag-tag constructions as described above.

We used strain 042AggRFlag, which contains a Flag-tag insertion at the 3′-end of the *aggR* gene and a kanamycin cassette, which confers resistance for selection, for a plasmid curing protocol. The protocol used was a modification of a previously reported (Hooper et al., 1984). Briefly, we started with an overnight culture of strain 042AggRFlag grown at 37°C in medium LB plus 50 μg ml^-1^ Km. Bacterial cells were re-inoculated (1:1000) in fresh LB medium during 10 consecutive days. Serial dilutions were then prepared from the 10th overnight culture in LB medium and bacterial cells were spread in LB plates supplemented with novobiocin (3,3 μg ml^-1^). Resistant colonies were tested for loss of Km^r^. Kanamycin sensitive colonies were subsequently tested for plasmid loss by PCR-detection of plasmid genes.

### Beta-Galactosidase Assay

β-Galactosidase activity measurements were performed as described by Miller (1992). The Student’s *t*-test was used to determine statistical significance, the values were obtained by using the GraphPad Prism 5 software. A *P*-value of less than 0.05 was considered significant.

### SDS-PAGE and Western Blotting

Protein samples were analyzed by SDS-PAGE at 12.5% (Sambrook, 2001). Proteins were transferred from the gels to PVDF membranes using the Trans-Blot Turbo system (Bio-Rad). Western blot analysis was performed with monoclonal antibody raised against the Flag-epitope (1:10.000 – Sigma) incubating 16 h at 4°C. Membranes were washed three times of 20 min each with PBS 0.2% Triton solution. Thereafter they were incubated with horseradish peroxidase-conjugated goat anti-mouse IgG (1:2500 – Promega) during 1 h at room temperature. Again, membranes were washed three times of 20 min with PBS 0.2% Triton solution and detection was performed by enhanced chemiluminescence using Quantity One software (Bio-Rad).

### Isolation of RNA

Bacterial cells were grown until OD_600__nm_ of 2.0. 5 ml of cells were then mixed with 0.2 volume of stop solution buffer (95% Ethanol, 5% Phenol), shaken and centrifuged (10 min, 6,000 × *g*). Bacterial pellets were subsequently frozen at -80°C until use. Total RNA was extracted from bacterial pellets using Tripure Isolation Reagent (Roche) according to the manufacturer’s instructions. Potential traces of DNA were removed by digestion with DNase I (Turbo DNA-free, Ambion), according to the manufacturer’s instructions. RNA concentration and RNA quality were measured using a Nano-Drop 1000 (Thermo Fisher Scientific).

### Quantitative Reverse Transcription-PCR (qRT-PCR)

Expression levels of *aggR*, *aafA*, *aafD*, *aatP*, and *aap* genes were determined by using real-time quantitative PCR. Briefly, 1 μg of previously isolated total RNA was reverse transcribed to generate cDNA using the High-capacity cDNA Reverse Transcription kit (Applied Biosystems) according to the manufacturer’s instructions. All samples within an experiment were reverse transcribed at the same time; the resulting cDNA was diluted 1:100 in nuclease-free water and stored in aliquots at -80°C until used. As a control, parallel samples in which reverse transcriptase was omitted from the reaction mixture, were run. Real-time PCR was carried out using Maxima SYBR green/ROX qPCR master mix (Thermo Scientific) and the ABI Prism 7700 sequence detection system (Applied Biosystems). Specific oligonucleotides complementary to the genes of interest were designed using primer3 software. The primers were named aggRRTFW/aggRRTRV, aafARTFW/aafARTRV, aafDRTFW/aafDRTRV, aatPRTFW/aatPRTRV, and aapRTFW/aapRTRV for *aggR*, *aafA*, *aafD*, *aatP*, and *aap* genes, respectively (see **Supplementary Table S1**, for the corresponding sequences). Relative quantification of gene expression of mutants versus wild-type strain was performed using the comparative threshold cycle (CT) method (Livak and Schmittgen, 2001). The relative amount of target cDNA was normalized using the *gapA* gene as an internal reference standard.

### Biofilm Quantification

Biofilm assay and quantification were performed as described (Sheikh et al., 2001) with some modifications. Briefly, bacterial cells were grown overnight in LB medium at 37°C and then, re-inoculated (1:1000) in fresh LB medium and incubated at 37°C until an OD_600_ of 2.0 was reached. These cultures were used to inoculate (1:100) wells of Nuclon Delta Surface plates (24 and 96 wells – Thermo Scientific) containing LB medium supplemented with glucose at 0.45% of final concentration. Plates were incubated at 37°C for 16 h. Biofilms were then washed twice with PBS and stained with 5% crystal violet. Biofilms were solubilized in 95% ethanol and quantified spectrophotometrically at 570 nm. The Student’s *t*-test was used to determine statistical significance. The values were obtained by using the GraphPad Prism 5 software. A *P*-value of less than 0.05 was considered significant.

## Results

### Effect of Temperature and Growth Phase on *aggR* Expression

**FIGURE 1:**
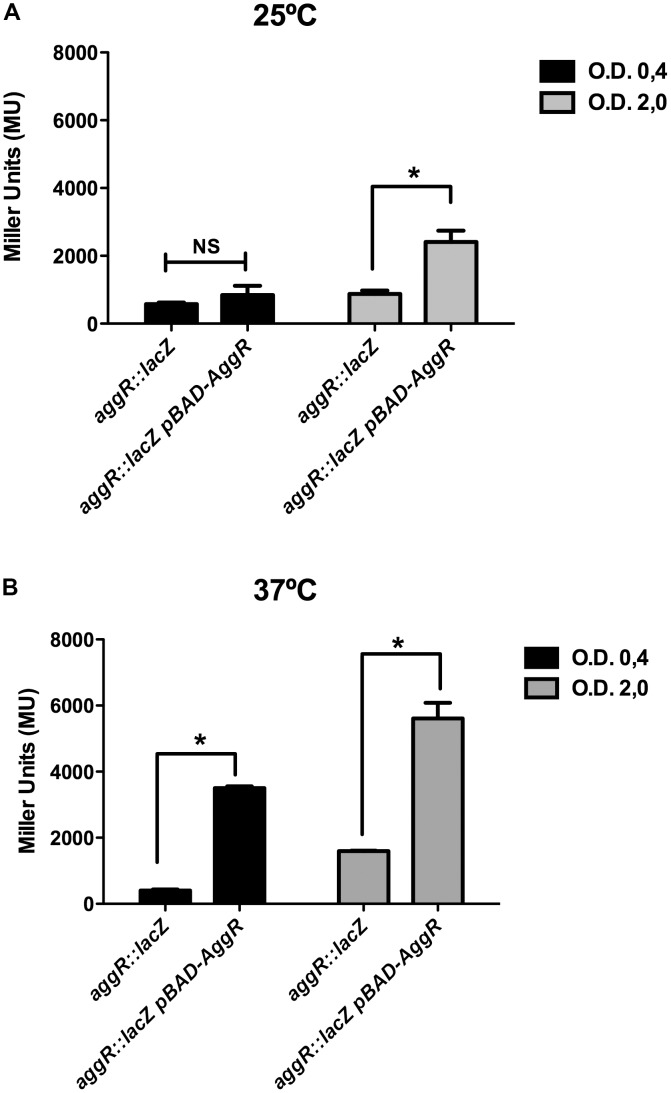
Effect of growth phase and temperature on *aggR* expression in strain 042. **(A)** β-Galactosidase activity in strain 042 cultures grown in LB medium with 0.45% of L-arabinose and collected at the exponential and early stationary growth phases. Cultures were grown at 25 **(A)** and 37°C **(B)**. Strain used was 042LCaggRLacZ without and with pBAD-AggR plasmid. Transformation of strain 042LCaggRLacZ with pBAD18 plasmid (empty vector) did not modify β-galactosidase activity. The data shown are the means and standard deviations of three independent experiments. Statistical analysis showed significative difference compared to the control (^∗^*P*-value < 0.0001; NS, non-significative).

The aim of this study was to gain insight into the regulation of the *aggR* gene in strain 042. Upon generating an *aggR::lacZ* transcriptional fusion, we studied first the effect of temperature and growth phase on the expression of this transcriptional activator. Taking into account that AggR positively modulates its expression (Morin et al., 2010) and that the transcriptional fusion generated by us disrupts AggR, we provided the AggR protein *in trans* by transforming plasmid pBAD-AggR in strain 042LCaggRLacZ. By doing this, we aimed to avoid that the lack of AggR would mask or alter the effect of different growth conditions on *lacZ* transcription. Strains 042LCaggRlacZ and 042LCaggRLacZ (pBAD-AggR) were grown in LB medium either at 25 or at 37°C, samples were taken both at the mid-logarithmic growth phase (OD_600_ of 0.4) and at the onset of the stationary phase (OD_600_ of 2.0), and beta-galactosidase activity was determined. The results obtained showed that there exists growth phase- and temperature-dependent regulation of *aggR* (**Figures 1A,B**). The presence of plasmid pBAD-AggR showed that, as predicted. AggR activates it own transcription.

### *aggR* Transcription Is Downregulated in a (p)ppGpp-Null Mutant Derivative of Strain 042

**FIGURE 2:**
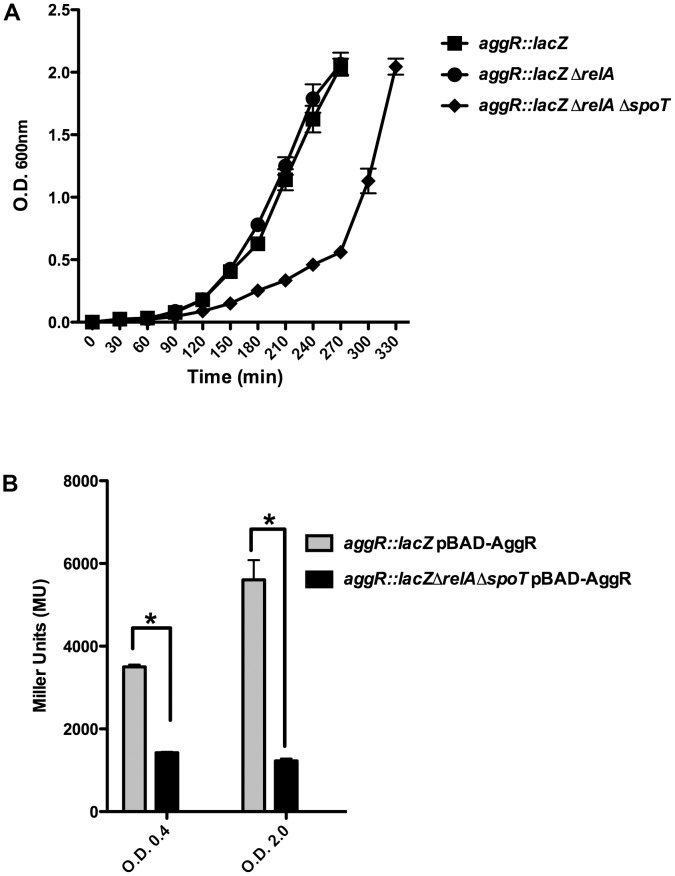
Expression of *aggR* is reduced in a (p)ppGpp^0^ derivative of strain 042. **(A)** Impact on growth rate of the *relA* and *relA spoT* derivatives of strain 042LCaggRlacZ. Growth curves of strains 042 *aggR::lacZ*, 042 *aggR::lacZ* Δ*relA* and *aggR::lacZ*Δ*relA*Δ*spoT*. Growth kinetics of the WT 042 strain (not shown) was identical to that of the 042 *aggR::lacZ* strain. **(B)** β-Galactosidase activity of strains 042LCaggRLacZ (pBAD-AggR) and 042LCaggRlacZrel AspoT (pBAD-AggR) grown at 37°C in LB medium with 0.45% of L-arabinose either to the exponential or to the early stationary growth phases. The data shown are the means and standard deviations of three independent experiments. Statistical analysis showed significant differences compared to the control (^∗^*P*-value < 0.0001).

**FIGURE 3:**
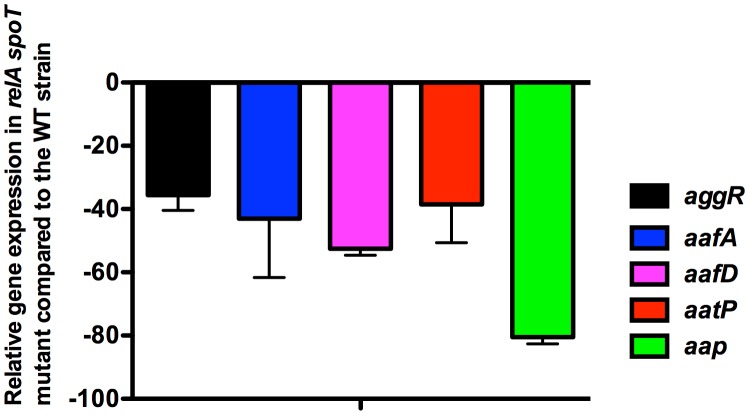
Effect of the double *relA spoT* mutation on transcription of *aggR*, and AggR-activated genes in strain 042. Fold change values (qRT-PCR values) of *aggR*, and AggR-activated genes *aafA*, *aafD*, *aatP*, and *aap* in strain 042 Δ*relA*Δ*spoT*. Expression of the corresponding genes in the 042 WT strain was considered as 1.0. The data shown are the means and standard deviations of three independent experiments are shown.

Upon obtaining a double 042 *relA spoT* mutant, we assessed whether lack of (p)ppGpp influences growth and *aggR* expression in strain 042. Strains 042LCaggRlacZ and 042LCaggRlacZrelAspoT were used for these studies. Although *aggR* expression is higher when cells grow in DMEM medium than in LB medium (Morin et al., 2010), we selected this latter in order to reduce the predicted impact of the double mutation *relA spoT* on the growth rate that cells harboring these mutations exhibit when growing in minimal media. Even growing in LB medium, the (p)ppGpp^0^ derivative of strain 042 (*aggR::lacZ*) showed a reduced growth rate (**Figure 2A**). To evaluate the role of (p)ppGpp on *aggR* expression, we measured first transcription of *aggR* as β-galactosidase activity. Again, both the 042 WT and the *relA spoT* mutant strains were transformed with plasmid pBAD-AggR. When compared with strain 042LCaggRlacZ, strain 042LCaggRlacZrelAspoT showed significantly reduced β-galactosidase levels (**Figure 2B**). The results obtained by using the *lacZ* transcriptional fusion were corroborated by specifically measuring *aggR* transcription by qRT-PCR (**Figure 3**). *aggR* expression is significantly downregulated in the (p)ppGpp^0^ mutant. We also used qRT-PCR to measure (p)ppGpp-dependent transcription of some of the AggR-activated genes, namely *aafA*, *aafD*, *aatP*, and *aap*. *aafA* and *aafD* genes encode the proteins responsible for the biogenesis of the AAF/II fimbriae (being AafA the major subunit and AafD a chaperone of AAF/II, respectively). AatP (an inner-membrane permease) belongs to the cluster responsible for secretion of the dispersin protein (AaP), which promotes dispersal of EAEC across the intestinal mucosa. As expected, due to the reduced expression of AggR, those genes altogether are downregulated in absence of (p)ppGpp (**Figure 3**).

### AggR and AafA Proteins Show a (p)ppGpp-Dependent Expression

**FIGURE 4:**
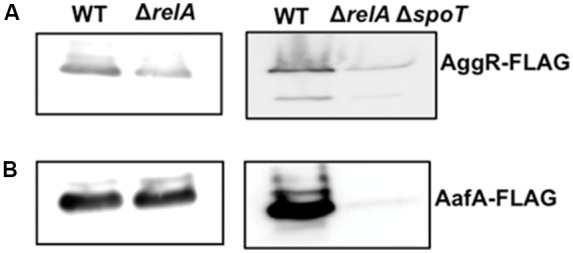
Expression of AggR and AafA proteins in strain 042 is reduced in the absence of (p)ppGpp. Immunodetection of AggR-Flag **(A)** and AafA-Flag **(B)** proteins in total cell extracts from cultures from strain 042 and its isogenic mutants Δ*relA* and Δ*relA*Δ*spoT*. Experiments were repeated three times. A representative experiment is shown.

To correlate transcriptional data with protein expression, *aggR* and *aafA* genes were Flag-tagged. Thereafter, Flag-tagged AggR and AafA proteins were immunodetected in total cell extracts, both in the WT 042 strain as well as in the corresponding *relA* and *relA spoT* derivatives. Culture conditions for AggR and AafA immunodetection were those used to measure *aggR* transcription [growth in LB medium at 37°C to the early stationary phase (OD_600_ of 2.0)]. The results obtained are in accordance with the transcriptional data obtained. The levels of both AggR and AafA proteins are reduced in the *relA spoT* derivative (**Figure 4**, see **Supplementary Figures S1**–**S3** to full Western blot membranes). The fact that they are not reduced in the *relA* mutant can be interpreted as *spoT* activity rendering (p)ppGpp levels high enough as to enable high-level expression of these proteins in strain 042.

### (p)ppGpp Is Required for Biofilm Formation in the EAEC Strain 042

**FIGURE 5:**
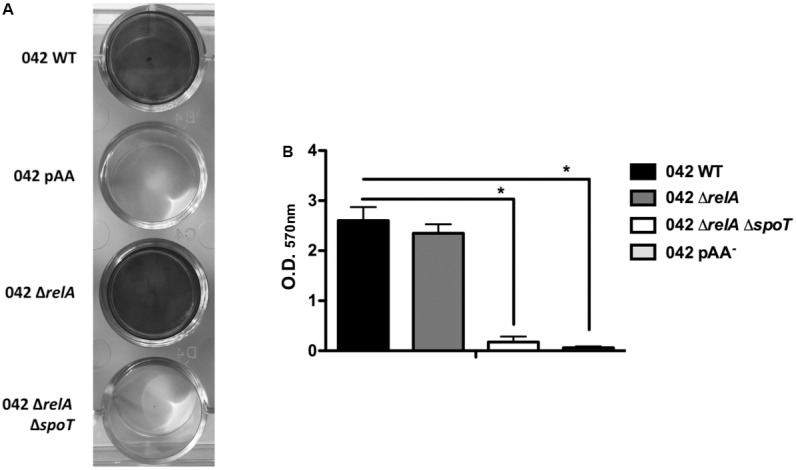
Biofilm formation in the 042 strain is directly dependent on (p)ppGpp. **(A)** Crystal violet stain of biofilms formed by strains 042, 042 pAA^-^, 042 Δ*relA*, and 042 Δ*relA*Δ*spoT.* **(B)** Biofilm quantification (OD_570__nm_). The data shown are the means and standard deviations of three independent experiments. Statistical analysis showed significant differences compared to the control (^∗^*P*-value < 0.0001).

**FIGURE 6:**
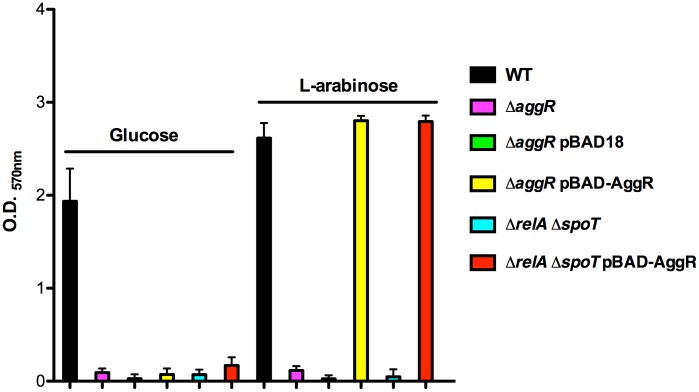
Biofilm formation in strain 042 requires both the AggR protein and physiological levels of (p)ppGpp. Quantification of biofilm formed by strain 042 and its *aggR* and *relAspoT* derivatives, in the presence and in the absence of plasmid pBAD-AggR. Cells were cultured in LB medium supplemented either with glucose or L-arabinose at 0.45%. The data shown are the means and standard deviations of three independent experiments.

It is well-established that *aggR* mutants are defective in biofilm formation in strain 042 (Sheikh et al., 2001). Downregulation of the major pilin subunit (AafA protein) is underlying that phenotype (Sheikh et al., 2001). Taking into account that, as shown above, AafA expression is dependent on proper (p)ppGpp levels, we hypothesized that biofilm formation should also be downregulated in a (p)ppGpp^0^ derivative of strain 042. To assess this, we measured biofilm formation in cells growing in LB-glucose (0.45% final concentration). Strains analyzed were WT 042, 042relAspoT, and 042pAA^-^. As expected, biofilm production is drastically reduced both in the (p)ppGpp^0^ mutant as well as in the WT strain lacking pAA2 plasmid (**Figures 5A,B**). We also decided to test whether expression *in trans* of the AggR protein can complement that phenotype. To do this, plasmid pBAD-AggR was transformed in strains WT 042, 042aggR, and 042relAspoT. Cells were grown in LB medium supplemented either with glucose (negative control) or L-arabinose (AggR expression), and biofilm formation was assessed in the different strains (**Figure 6**). When cells were grown in conditions leading to specific AggR expression (LB plus L-arabinose), the presence of plasmid pBAD-AggR induced biofilm formation both in strains 042aggR and 042relAspoT, thus providing evidence for AggR being required for biofilm formation, and correlating (p)ppGpp and biofilm formation via AggR (**Figure 6**).

## Discussion

**FIGURE 7:**
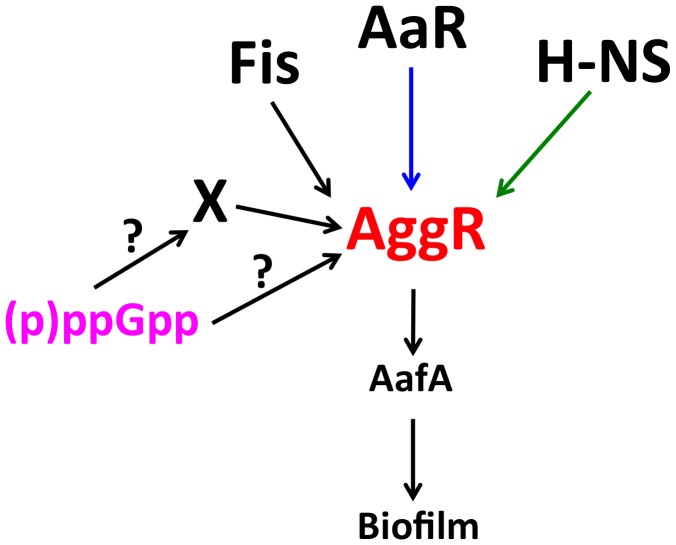
Diagram showing the regulatory cascade influencing AggR expression. AggR is a key virulence regulator in EAEC. Downstream-regulated virulence factors include the AafA pilin subunit. FIS and H-NS proteins respectively induce and repress *aggR* transcription (black and green arrows, respectively). AaR protein post-transcriptionally represses AggR protein expression (blue arrow). We show in this work that the (p)ppGpp alarmone is a key factor regulating either directly or indirectly expression of *aggR* and AggR-activated genes.

We bring in this paper new information about the physiological inputs that dictate AggR expression (and hence virulence expression) in the EAEC strain 042. As it happens with many other virulence factors in *E. coli* and other pathogens infecting warm-blooded hosts, virulence expression requires temperatures close to that of their hosts. Temperature-dependent expression of AggR is consistent with the reported effect of the nucleoid-associated protein H-NS repressing its expression (Morin et al., 2010) (**Figure 7**). We also found that there is growth-phase dependent expression of *aggR* when cells grow in LB medium. A significant increase in *aggR* transcription occurs when cells enter the stationary phase (OD_600_ of 2.0). Other authors have shown that *aggR* is maximally expressed during the exponential growth phase (Morin et al., 2010) when cells were grown at 37°C in DMEM medium plus glucose. Most likely, the nature of these growth media (rich versus minimal) is underlying these differences.

There exist several examples of (p)ppGpp playing a central role in bacterial persistence and virulence (Pizarro-Cerdá and Tedin, 2004; Song et al., 2004; Thompson et al., 2006; Dalebroux et al., 2010; Gaca et al., 2015). We show in this report this is also the case for the expression of the AggR regulator in the EAEC strain 042. A (p)ppGpp^0^ mutant shows reduced *aggR* transcription and fails to induce it when cells enter the stationary phase, conditions that can be correlated with high (p)ppGpp levels. A*ggR* expression is similar in the 042 WT strain and in its *relA* derivative. This suggests that *spoT* (p)ppGpp synthase activity can generate alarmone levels high enough as to maintain high AggR expression. As predicted because of the modulatory role of AggR, lack of (p)ppGpp can also be correlated with reduced expression of AggR modulated genes, such as *aafA* (see **Supplementary Figure S4**). (p)ppGpp effects can occur by a variety of mechanisms (as reviewed in Magnusson et al., 2005; Hauryliuk et al., 2015; Steinchen and Bange, 2016). A question that remains to be answered is if (p)ppGpp modulates *aggR* transcription because its direct effect on RNA polymerase, or indirectly, via (p)ppGpp mediated-induction of RpoS (**Figure 7**).

(p)ppGpp-dependent upregulation of *aggR* and its regulatory cascade can be correlated with a virulence phenotype. We could abolish biofilm formation in strain 042 both by (i) generating a (p)ppGpp^0^ mutant, (ii) by knocking out the *aggR* gene, and (iii) by curing this strain of the pAA2 plasmid. Biofilm formation can be restored both in an *aggR* and in a *relA spoT* mutant by providing *in trans* the AggR protein, thus supporting the hypothesis that (p)ppGpp-dependent AggR levels are critical for biofilm formation in strain 042. Previous reports have shown that (p)ppGpp influences the phase-variation of *fim* promoter in *E. coli*, suggesting a dependence upon (p)ppGpp in type 1 fimbriation and consequently in biofilm formation in both *E. coli* K12 and uropathogenic *E. coli* strains (Aberg et al., 2006). We show here that the relationship between (p)ppGpp and biofilm formation in the EAEC strain 042 occurs, instead of via type 1 fimbriae, via AggR and AAF/II fimbriae.

The fact that our results show a direct correlation between (p)ppGpp, virulence gene expression (e.g., AafA protein) and biofilm formation in strain 042 can be of interest to find out new strategies to combat bacterial infections caused by EAEC. Taking into account that several from these strains can also display a phenotype of multiple antibiotic resistance, combatting infections caused by these strains requires the development of new strategies. Targeting the bacterial stringent response has been studied in the last years as a new approach to combat bacterial infections and biofilm formation (de la Fuente-Núñez et al., 2014; Gaca et al., 2015). A synthetic peptide was recently proposed to specifically disrupt biofilms by inhibiting the stringent response via direct interaction with (p)ppGpp (Andresen et al., 2016). The results presented here suggest that these approaches could also be used to combat multiresistant EAEC-mediated infections.

## Author Contributions

MH and AJ conceived and designed the experiments and wrote the manuscript. MH, AP, JE, and MB performed the experiments.

## Conflict of Interest Statement

The authors declare that the research was conducted in the absence of any commercial or financial relationships that could be construed as a potential conflict of interest.

## Supplementary Material

FIGURE S1 Full Western blot membrane showing the AggR-Flag and AafA-Flag expression corresponding to **Figure 4**, lanes 1 and 3 WT, lanes 2 and 4 *relA* mutant.

FIGURE S2 Full Western blot membrane showing the AggR-Flag expression corresponding to **Figure 4**, lane 1 WT and lane 2 *relA spoT* mutant.

FIGURE S3 Full Western blot membrane showing the AafA-Flag expression corresponding to **Figure 4**, lane 1 WT and lane 2 *relA spoT* mutant.

FIGURE S4 Complementation of the double *relA spoT* mutantion by providing SpoT *in trans* by using the pBR322-SpoT plasmid. **(A)** M9 minimal medium agar plate showing that the ppGpp^0^ strain (Δ*relA*Δ*spoT*) is able to grow in M9 medium in the presence of pB322-SpoT plasmid. **(B)** Western blot showing that AafA-Flag protein expression is increased in the Δ*relA*Δ*spoT* mutant when SpoT is provided *in trans*. (1) 042 WT (wild-type strain), (2) 042AafAFlag (wild-type strain expressing AafA-Flag), (3) 042AafAFlagrelAspoT [(p)ppGpp^0^ derivative of 042 strain expressing AafA-Flag], (4) 042AafAFlagrelAspoT pBR322 (AafA-Flag ppGpp^0^ strain with control vector), and (5) 042AafAFlagrelAspoT pBR322-SpoT (AafA-Flag ppGpp^0^ strain expressing SpoT *in trans*).

TABLE S1 Oligonucleotides used in this work.
