# Supplementary figures and images for: Stringent Response and AggR-Dependent Virulence Regulation in the Enteroaggregative Escherichia coli Strain 042
Source: Front Microbiol. 2018 Apr 10;9:717. doi: 10.3389/fmicb.2018.00717 (PMC5902536; doi:10.3389/fmicb.2018.00717)

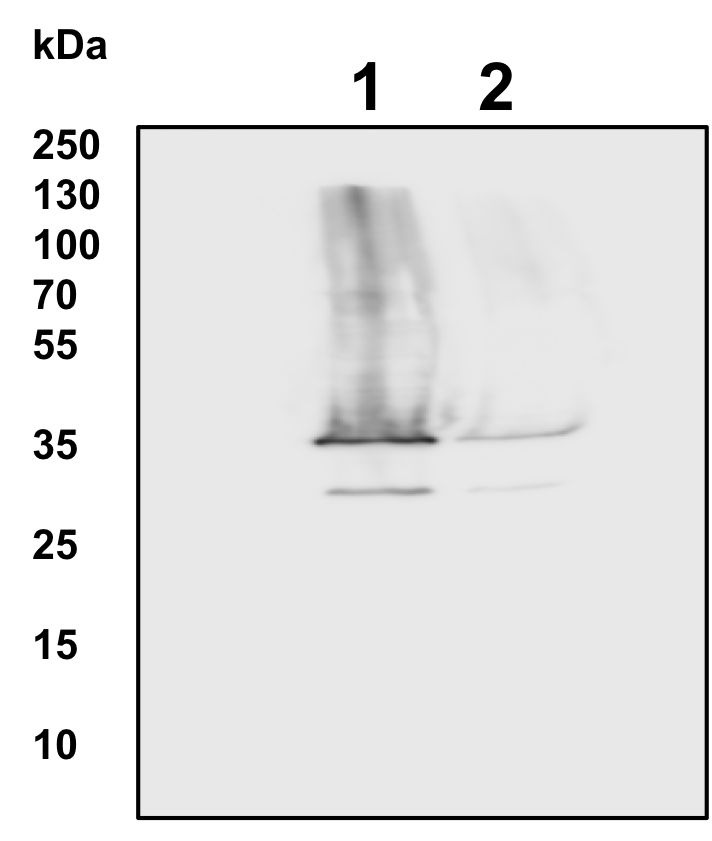

Supplement: FIGURE S1 — Full Western blot membrane showing the AggR-Flag and AafA-Flag expression corresponding to Figure 4, lanes 1 and 3 WT, lanes 2 and 4 relA mutant. [file Image_2.TIF]

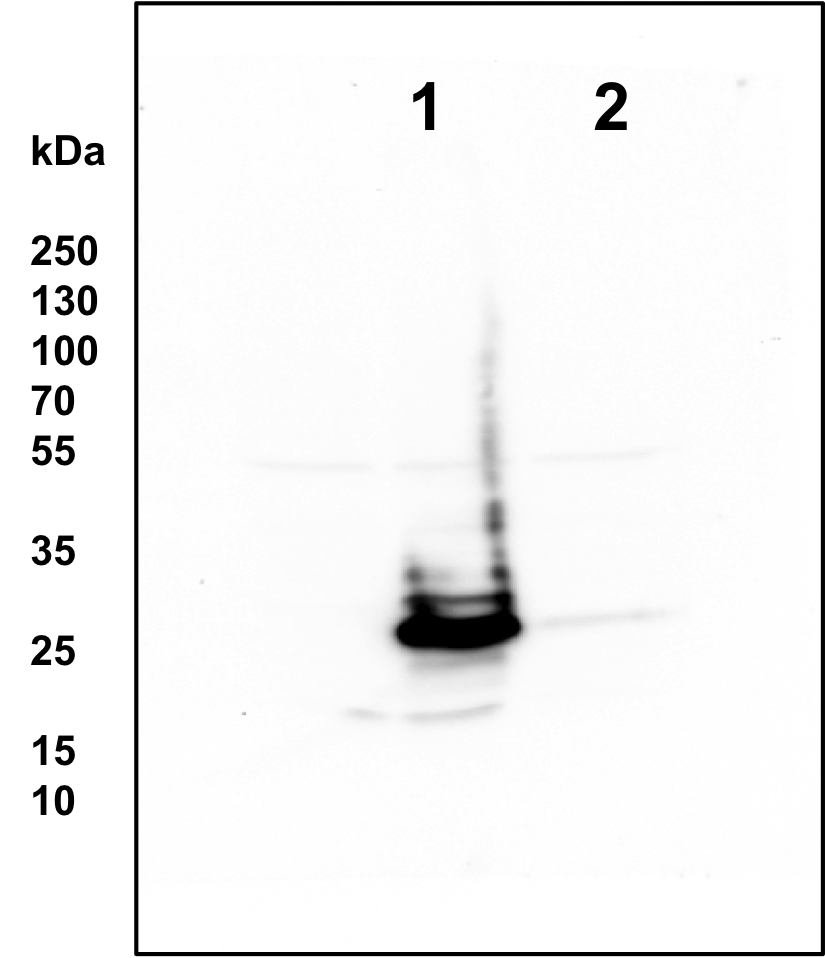

Supplement: FIGURE S2 — Full Western blot membrane showing the AggR-Flag expression corresponding to Figure 4, lane 1 WT and lane 2 relA spoT mutant. [file Image_3.TIF]

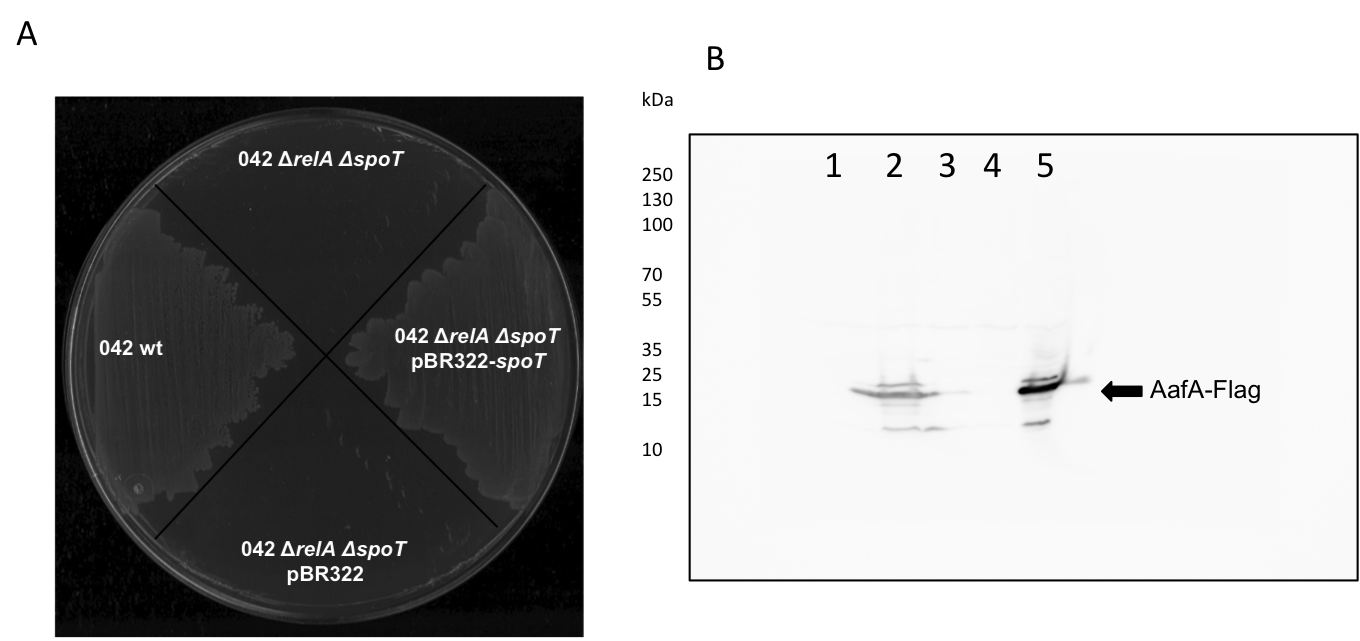

Supplement: FIGURE S3 — Full Western blot membrane showing the AafA-Flag expression corresponding to Figure 4, lane 1 WT and lane 2 relA spoT mutant. [file Image_4.TIF]

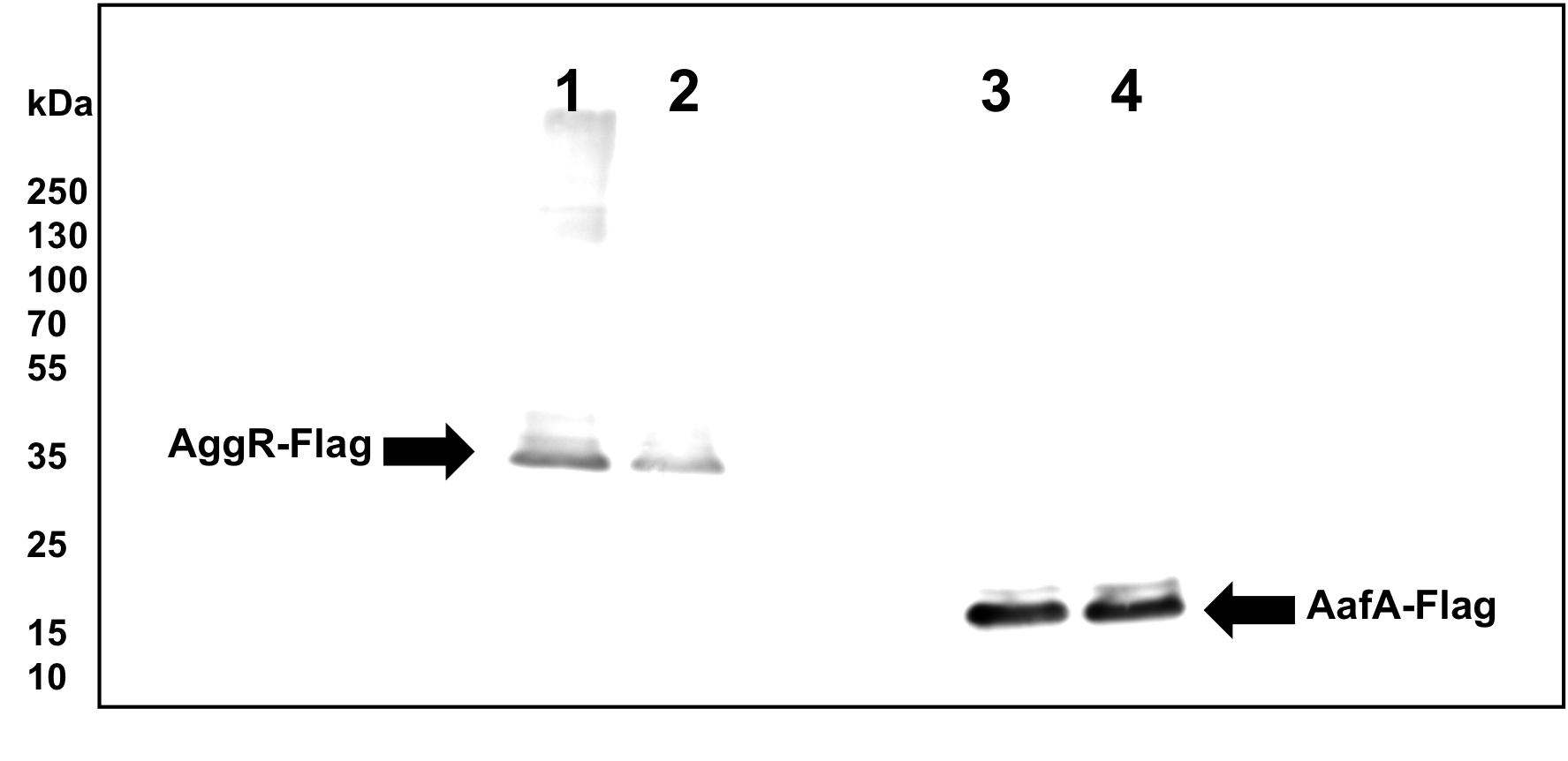

Supplement: FIGURE S4 — Complementation of the double relA spoT mutantion by providing SpoT in trans by using the pBR322-SpoT plasmid. (A) M9 minimal medium agar plate showing that the ppGpp0 strain (ΔrelAΔspoT) is able to grow in M9 medium in the presence of pB322-SpoT plasmid. (B) Western blot showing that AafA-Flag protein expression is increased in the ΔrelAΔspoT mutant when SpoT is provided in trans. (1) 042 WT (wild-type strain), (2) 042AafAFlag (wild-type strain expressing AafA-Flag), (3) 042AafAFlagrelAspoT [(p)ppGpp0 derivative of 042 strain expressing AafA-Flag], (4) 042AafAFlagrelAspoT pBR322 (AafA-Flag ppGpp0 strain with control vector), and (5) 042AafAFlagrelAspoT pBR322-SpoT (AafA-Flag ppGpp0 strain expressing SpoT in trans). [file Image_1.TIF]
