# Supplementary material for: Stringent Response and AggR-Dependent Virulence Regulation in the Enteroaggregative Escherichia coli Strain 042
Source: Front Microbiol. 2018 Apr 10;9:717. doi: 10.3389/fmicb.2018.00717 (PMC5902536; doi:10.3389/fmicb.2018.00717)
Supplement: TABLE S1 — Oligonucleotides used in this work. [file Table_1.DOC]

**Stringent response and AggR-dependent virulence regulation in the enteroaggregative *Escherichia coli* strain 042**

Hüttener^1^, M., Prieto^1^, A., Espelt^1^ J., Bernabeu^1^, M. and Juárez^1,2^ A*

^1^Department of Genetics, Microbiology and Statistics, University of Barcelona, Barcelona, Spain

^2^Institute for Bioengineering of Catalonia, The Barcelona Institute of Science and Technology, Barcelona, Spain

*Corresponding author: Antonio Juárez ([ajuarez@ub.edu)](mailto:ajuarez@ub.edu). Department of Genetics, Microbiology and Statistics. Faculty of Biology. University of Barcelona. Av. Diagonal, 643. 08028. Barcelona, Spain. Phone (+34) 934034624. Fax (+34) 934034629.

Keywords: (p)ppGpp, AggR, EAEC, Biofilm, AAF/II

Running title: (p)ppGpp and virulence in EAEC

**Supplementary Table 1.** Oligonucleotides used in this work.

| **Oligonucleotide** | **Sequence (5’-3’)** | **Purpose** |
| --- | --- | --- |
| lacZ042P1 | TGAGCGGATAACAATTTCACACAGGATACAGCTATGACTATGGTGTAGGCTGGAGCTGCTTC | *lacZ* deletion |
| lacZ042P2 | AAATACGGGCAGACATGGCCTGCCCGGTTATTATTATTTTTGCATATGAATATCCTCCTTAGT | *lacZ* deletion |
| lacZ042P1Up | CGTTGGCAGATTCTTTAATGC | *lacZ* deletion confirmation |
| lacZ042P2Down | CCGGTAATAATCCACAGCAGG | *lacZ* deletion confirmation |
| cat042P1 | GAGTTATCGAGATTTTCAGGAGCTAAGGAAGCTAAAATGGAGGTGTAGGCTGGAGCTGCTTC | *cat* deletion |
| cat042P2 | AGGCGTTTAAGGGCACCAATAACTGCCTTAAAAAAATTACGCCATATGAATATCCTCCTTAGT | *cat* deletion |
| cat042P1Up | GAAAATGAGACGTTGATCGGC | *cat* deletion confirmation |
| cat042P2Down | CATAAGCGGCTATTTAACGAC | *cat* deletion confirmation |
| aggR P1 | ACATTTTTTTCATGTGAGAATGATATGAAATTAAAACAAAACGTGTAGGCTGGAGCTGCTTC | *aggR* deletion |
| aggR P2 | TTATTGGCTTTTAAAATAAGTCAAGAATTGTTTTGGTGTTATCATATGAATATCCTCCTTAGT | *aggR* deletion |
| aggR P1Up | GCTGCAATTAAGATACAACCCC | *aggR* deletion confirmation |
| relA P1 | TGCCGATTTCGGCAGGTCTGGTCCCTAAAGGAGAGGACGATGGTTGCGGTGTAGGCTGGAGCTGCTTC | *relA* deletion |
| relA P2 | TCGCTGGATACGGCTGACGCCATATCCAGCTATATAAGACTAACTCCCCATATGAATATCCTCCTTAGT | *relA* deletion |
| relA P1Up | ACTGGAACCTATTCGTATAG | *relA* deletion confirmation |
| relA P2Down | GCGCCCTTCTTCCTGAGCCA | *relA* deletion confirmation |
| spoT P1 | ATTGCTGAAGGTCGTCGTTAATCACAAAAGCGGGTCGCCCTTGTATCTGGTGTAGGCTGGAGCTGCTTC | *spoT* deletion |
| spoT P2 | TTCGCAGATACGTGCATAACGTGTTGGGTTCATAAAACATTAATTTCGCATATGAATATCCTCCTTAGT | *spoT* deletion |
| spoT P1Up | GATGCAGGTAGGCGGAAAGG | *spoT* deletion confirmation |
| spoT P2Down | GGCCTTTCAGATGAGCGACG | *spoT* deletion confirmation |
| kt | CGGCCACAGTCGATGAATCC | Km cassette confirmation |
| k2 | CGGTGCCCTGAATGAACTGC | Km cassette confirmation |
| CAT-C1 | CCTTGTCGCCTTGCGTATAA | Cm cassette confirmation |
| CAT-C2 | CCTACCTGTGACGGAAGATC | Cm cassette confirmation |
| lacZR | GATGACCTGCAAGGCGATTA | *lacZ* confirmation insertion |
| aggREcoRI185 | CGGAATTCTTTTTTTCATGTGAGAATGATATG | AggR cloning pBAD18 |
| aggRXbaI183 | GCTCTAGATTATTGGCTTTTAAAATAAGT | AggR cloning pBAD18 |
| aggR3xP1 | CATAACACCAAAACAATTCTTGACTTATTTTAAAAGCCAAGACTACAAAGACCATGACGG | FLAG insertion |
| aggR3xP2 | TTTATAGCAATCTCAAATAATGATATGAAACATGTTTTTATTGGCATATGAATATCCTCCTTAG | FLAG insertion |
| aggR3xP1Up | GCTTGATAACTCATATCAGATATC | FLAG confirmation |
| aggR3xP2Down | CTCCTGTTCAGCTACTGACG | FLAG confirmation |
| aafA3xP1 | AATATATGAAGTAGTATTAAATGCTGAGCTTGTGACAAATGACTACAAAGACCATGACGG | FLAG insertion |
| aafA3xP2 | CCAAAAGTTATTATATTGTCACAAGCTCAGCATTTAATTTCATATGAATATCCTCCTTAG | FLAG insertion |
| aafA3xP1Up | GCCTCTCCTAGGTTTTCTTAC | FLAG confirmation |
| aafA3xP2Down | CACTATCAATAAGTTGGAGTC | FLAG confirmation |
| aggRRTFW | AGGAAAAGGCTTGAGTCAGAGT | qPCR |
| aggRRTRV | AGAATTGTTTTGGTGTTATGCCA | qPCR |
| aafARTFW | GGAGCGCAAATATCGACCTG | qPCR |
| aafARTRV | GCCAGAGTGAATCCTGCTGA | qPCR |
| aafDRTFW | CCCGCGTGGTTTATAATCAACA | qPCR |
| aafDRTRV | CTCCAATTCTGACGACTCGC | qPCR |
| aatPRTFW | AACGGAATTGACAAGCTGGG | qPCR |
| aatPRTRV | GCTTGCTTTCAATCCAAGGC | qPCR |
| aapRTFW | GCGGTTGGAACGCAGATAAT | qPCR |
| aapRTRV | TGAAGTTGCTTGTTGTGCCA | qPCR |
| spoT042pbr322 ECORI fw5 | CGGAATTCCGCGCTCGTCAGATGCAGGTA | SpoT cloning pBR322 |
| spoT042 pbr322 BAMHI rev 3 | CGGGATCCCGAAGCCATGGTGCGCATCCG | SpoT cloning pBR322 |
